# Hepatic Arterioportal Fistula: A Curable Cause of Portal Hypertension in Infancy

**DOI:** 10.1155/1997/58026

**Published:** 1997

**Authors:** J. S. Billing, N. V. Jamieson

**Affiliations:** Department of Surgery Addenbrooke's Hospital Cambridge UK

## Abstract

Hepatic arterioportal fistulae are a rare cause of portal hypertension. The case is reported of a twoyear old girl with a congenital arterioportal fistula, who presented with splenomegaly and ascites. Colour doppler ultrasound showed a large shunt between the left hepatic artery and a branch of the left portal vein, producing a reversal of flow in the main portal vein. She was treated by a formal left hemihepatectomy, which has been successful in eliminating the fistula and its consequent portal hypertension in the long term. The literature regarding arterioportal fistulae and their treatment is reviewed.


HPB Surgery, 1997, Vol. 10, pp. 311-314
Reprints available directly from the publisher
Photocopying permitted by license only

© 1997 OPA (Overseas Publishers Association)
Amsterdam B.V. Published in The Netherlands
by Harwood Academic Publishers
Printed in India

*Case Report*

### Hepatic Arterioportal Fistula: A Curable Cause of Portal Hypertension in Infancy

J. S. BILLING[a] and N. V. JAMIESON[b]

[a]*Senior House Officer,* [b]*Consultant Surgeon, Department of Surgery, Addenbrooke's Hospital, Cambridge*

*(Received in final form 7 November 1996)*

**Hepatic arterioportal fistulae are a rare cause of portal hypertension. The case is reported of a two-year old girl with a congenital arterioportal fistula, who presented with splenomegaly and ascites. Colour doppler ultrasound showed a large shunt between the left hepatic artery and a branch of the left portal vein, producing a reversal of flow in the main portal vein. She was treated by a formal left hemihepatectomy, which has been successful in eliminating the fistula and its consequent portal hypertension in the long term. The literature regarding arterioportal fistulae and their treatment is reviewed.**

*Keywords:* Portal hypertension, arterioportal fistula, congenital, liver resection

### INTRODUCTION

Fistulae between the hepatic artery and portal vein are an uncommon but potentially curable cause of portal hypertension. Shunting of hepatic arterial blood into the portal circulation most commonly occurs as a result of an acquired arterioportal fistula [1]. Congenital arterioportal fistulae are rare, with only a small number of case reports describing this entity in children [2-4]. We report here the sixth case of a congenital intrahepatic fistula between the hepatic artery and the portal vein in a child, and the first such case to be successfully treated by hemihepatectomy.

### CASE REPORT

R. S. is a female child born spontaneously at term who was noted to have the facies of Down's syndrome. Chromosomal analysis confirmed the presence of trisomy 21 and she was followed up regularly in the Child Development Centre. The only other abnormality apparent early in life was a secundum atrial septal defect which did not cause any significant haemodynamic compromise. She was otherwise generally in good health.

On review in the Child Development Centre at 9 months of age she was noted to be pale, with

Correspondence to: N. V. Jamieson, Department of Surgery, Addenbrooke's Hospital, Hills Road, Cambridge CB2 2QQ, U.K.

an easily palpable spleen extending 4 cm below the costal margin. A reducable right inguinal hernia had also recently been noted by the mother. Haematological examination showed a haemoglobin of 9.3 g/dL, with a white cell count of 3.7 and a platelet count of 152. The blood film revealed variable red cell sizes with polychromasia but no blasts. A planned hernia repair was cancelled because of the anaemia.

When she was 15 months old, she presented with a two week history of increasing abdominal girth, and her inguinal hernia, which had initially been noted only intermittently, was now present continually. Clinical examination revealed the presence of ascites, with a fluid thrill evident. At this stage she was still anaemic, but her liver function tests were normal. An ascitic tap was performed, which showed clear transudate fluid with no malignant cells, Ultrasonography demonstrated marked ascites with splenomegaly but a normal looking liver. Colour doppler ultrasound revealed a large arteriovenous shunt (2 cm in diameter) between the left hepatic artery and the left branch of the portal vein, which was seen to produce an oscillating reversal of flow in the main portal vein. This arteriovenous shunt was sufficiently large to explain the observed portal hypertension, resulting in splenomegaly and ascites.

She was started on spironolactone therapy for the ascites. Angiography was attempted in order to investigate this arteriovenous malformation, but a radiologist skilled in paediatric interventional techniques found it impossible to catheterise either femoral artery. A decision was therefore made a proceed to laparotomy.

At operation a dilated arteriovenous malformation was identified in the left hemi liver (Fig. 1), just to the right of the falciform ligament extending down into the umbilical fissure without a clear plane between the malformation and the division of the left hepatic vessels into their branches to segments II, III and IV. An intraoperative angiogram via the gastroduodenal artery confirmed that the right hepatic circulation was normal with gross abnormality of the left sided system in relation to the visible abnormality in segment IV. A biopsy of the liver was taken and confirmed normal histology. Local excision was not felt to be possible given the extent and the site of arteriovenous malformation and a formal left hemihepatectomy was undertaken, the raw liver surface being sealed with fibrin glue and collagen. Bilateral inguinal hernias were closed from inside the abdomen.

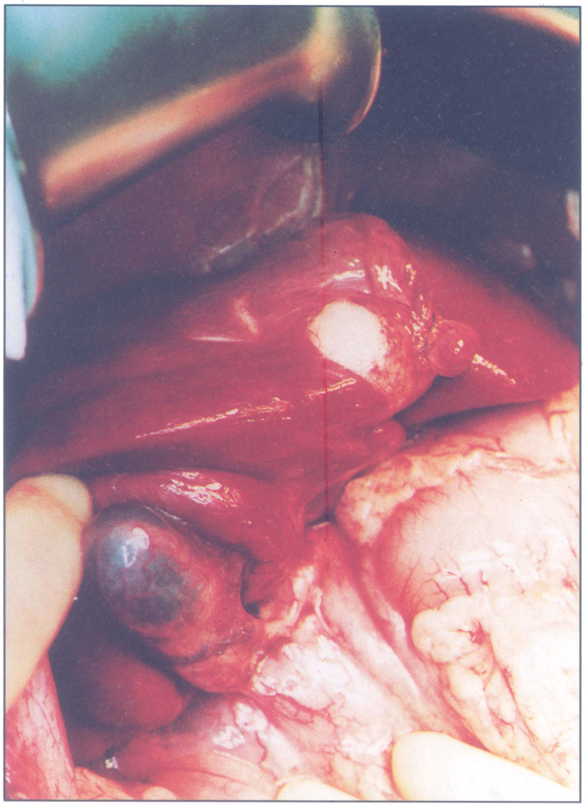

FIGURE 1 Operative photograph showing the arterioportal fistula in the left lobe of the liver, immediately to the right of the falciform liagment. (See Color Plate III).

Histological examination of the resected left liver demonstrated dilated blood vessels near the resection margin, which were filled with fresh blood clot. The maximum diameter of the lumen was 1.5 cm. On microscopy this was seen to be a large dilated vein with marked fibro-intimal thickening and features of an arteriovenous malformation, Post-operatively, ultrasound scans showed normal doppler traces of the right hepatic artery, right portal vein and right hepatic vein. In particular, the arterialisation and reversal of flow in the portal vein observed preoperatively was no longer present.

The early postoperative course was complicated by a bile leak from the ligated stump of the left hepatic duct. This was dealt with a second laparotomy eight days after the hemihepatectomy, when a T-tube was inserted into the residual duct. The subsequent postoperative course was uneventful and the T-tube was removed after three months, following a satisfactory T-tube cholangiogram. Ultrasonography six months after her surgery showed that the right hemi liver had hypertrophied and the spleen had decreased in size. There was no ascites and normal portal vein and hepatic artery traces were identified with doppler. It is now over four years since her liver resection and there has been no evidence of recurrence of her portal hypertension.

### DISCUSSION

There are a number of reported causes of arterioportal fistulae, which may be either intra- or extra-hepatic. Blunt and penetrating liver trauma, including iatrogenic injury, are the most common [1]. There are a number of reports of such fistulae occurring as a result of percutaneous liver biopsy [1, 5]. Intrahepatic arterioportal fistulae were found in as many as five out of 65 consecutive cases of blunt hepatic trauma [6], but most of these were small peripheral fistulae with low flow and closed spontaneously. In Tanaka's series only one case (a central fistula) persisted and required embolisation [6]. Erosion of splanchnic artery aneurysms into the portal circulation is another relatively common cause of fistulae [7] and many of these will occur in extrahepatic locations. Other causes of arterioportal fistulae are rare, and include liver turmours [8], hepatic amyloidosis [9] and much less commonly congenital cases.

Congenital shunts between the hepatic artery and portal vein are rare lesions, as evidenced by the small number of cases previously reported. Routh *et al.* could find only three previous cases when they reviewed the literature in 1992 [3], and there has been only one subsequent case reported from Berlin [4]. The case reported here is also remarkable in that surgical treatment has been successful in eliminating the source of portal hypertension in the long term.

The presentation of hepatic arterioportal fistulae depends particularly on the flow through the shunt and thereby its haemodynamic consequences. Those with small shunts may present as incidental findings at surgery for other reasons, as a chance finding at post mortem examination, or they may be detected in asymptomatic patients when a bruit and thrill are noted over the liver. When there is a clinical presentation it is most commonly characterised by the development of ascites or haemorrhage from oesophageal varices, as a result of portal hypertension [3, 8–10]. An unusual manifestation of portal hypertension with which some of these fistulae have presented is mesenteric vascular congestion, resulting in symptoms of abdominal pain and diarrhoea [1, 11].

It is of note that fistulae between the hepatic artery and portal vein tend not to produce heart failure. This is in marked contrast to systemic arteriovenous shunts, which are a well recognised cause of high output cardiac failure. Foley has artributed this difference to the presence of the hepatic sinusoids lying between the vascular lesion and the right heart, which impose a significant restriction on flow [12]. An alternative explanation is that fistulae between the hepatic artery and portal vein present earlier in their natural history due to the adverse effects of portal hypertension, even when the flow through the fistula is still relatively modest.

The rationale behind treatment of arterioportal fistulae is either to overcome existing portal hypertension or to prevent its development. It has been demonstrated that some small arterioportal fistulae can regress spontaneously [12]. However, those shunts which are producing portal hypertension tend to persist and require treatment. The most direct form of treatment is formal suture ligation of the fistula itself, but this tends to be restricted to extrahepatic lesions. An alternative is to ligate the vessel or vessels feeding the fistula [2, 3], although this has not always left the patient free from recurrence of portal hypertension as collateral vessel develop and allow the fistula to enlarge once more. A far less invasive approach has been transcatheter arterial embolisation of these arterioportal fistulae [3, 5, 6] which has been used successfully in some cases.

For those intrahepatic arterioportal fistulae confined to one hemi liver or segment of the liver, hepatic resection offers a more definitive form of treatment [10, 13]. Liver resection is a rather more extensive procedure than the other approaches discussed, with greater risk of attendant morbidity, but it does avoid the problem of recurrent portal hypertension which has been encountered when less radical techniques which have been employed. It also provides a valuable second line of treatment when a less aggressive approach has been unsuccessful. We believe the case reported here to the first example of portal hypertension in a child caused by a hepatic arterioportal fistula to be successfully treated by hemihepatectomy.

### *References*

[1] Strodel, W. E., Eckhauser, F. E., Lemmer, J. H., Whitehouse, W. M. and Williams, D. M. (1987). Presentation and perioperative management of arterioportal fistulas. *Archives of Surgery*, **122**, 563–71.

[2] Helikson, M. A., Shapiro, D. L. and Seashore, J. H. (1977). Hepatoportal arteriovenous fistula and portal hypertension in an infant. *Paediatrics*, **60**, 921–4.

[3] Routh, W. D., Keller, F. S., Cain, W. S. and Royal, S. A. (1992). Transcatheter embolization of a high-flow congenital intrahepatic arterial-portal venous malformation in an infant. *Journal of Paediatric Surgery*, **27**, 511–4.

[4] Gorenflo, M., Waldschmidt, J., Bein, G., Flocken, W. and Vogel, M. (1993). Arterioportal fistula in infancy. *Journal of Paediatric Gastroenterology and Nutrition*, **16**, 87–9.

[5] Isik, F. F., Greenfield, A. J., Guben, J., Birkett, D. and Menzoian, J. O. (1989). latrogenic arterioportal fistulae: diagnosis and management. *Annals of Vascular Surgery*, **3**, 52–5.

[6] Tanaka, H., Iwai, A., Sugimoto, H., Yoshioka, T. and Sugimoto, T. (1991). Intrahepatic arterioportal fistula after blunt hepatic trauma: case reports. *Journal of Trauma*, **31**, 143–6.

[7] Vanway, C. W., Crane, J. M., Riddell, D. H. and Foster, J. H. (1971). Arteriovenous fistula in the portal circulation. *Surgery*, **70**, 876–90.

[8] Granov, A. M., Tarazov, P. G. and Ryzhkov, V. K. (1992). Arterioportal fistulas in liver tumors: prognosis in relation to treatment. *HPB Surgery*, **5**, 87–94.

[9] Aramaki, T., Terada, H., Okumura, H., Tsutsui, H., Fujita, S., Tajiri, T., Ohya, T. and Tajima, H. (1989). Portal hypertension secondary to intrahepatic arterioportal shunt in primary amyloidosis: a case report. *Gastroenterology of Japan*, **24**, 4103.

[10] OishiA. J., NagorneyD. M. and CherryK. J. (1993). Portal hypertension, variceal bleeding and high output cardiac failure secondary to an intrahepatic arterioportal fistula. *HPB Surgery*, **7**, 53–9.

[11] Gryboski, J. D. and Clemett, A. (1967). Congenital hepatic artery aneurysm with superior mesenteric artery insuciency: a steal syndrome. *Paediatrics*, **39**, 344.

[12] Foley, W. J., Turcotte, J. G., Hoskins, P. A., Brant, R. L. and Ause, R. G. (1971). Intrahepatic arteriovenous fistulas between the hepatic artery and portal vein. *Annals of Surgery*, **174**, 849–55.

[13] Shumacker, H. B. and Waldhausen, J. A. (1961). Intrahepatic arteriovenous fistula of hepatic artery and portal vein. *Surgery. Gynecology and Obstetrics*, **112**, 497–501.

